# Current Knowledge of MicroRNAs (miRNAs) in Acute Coronary Syndrome (ACS): ST-Elevation Myocardial Infarction (STEMI)

**DOI:** 10.3390/life11101057

**Published:** 2021-10-08

**Authors:** Daniela Maria Tanase, Evelina Maria Gosav, Anca Ouatu, Minerva Codruta Badescu, Nicoleta Dima, Ana Roxana Ganceanu-Rusu, Diana Popescu, Mariana Floria, Elena Rezus, Ciprian Rezus

**Affiliations:** 1 Department of Internal Medicine, “Grigore T. Popa” University of Medicine and Pharmacy, 700115 Iasi, Romania; tanasedm@gmail.com (D.M.T.); ank_mihailescu@yahoo.com (A.O.); codruta.badescu@gmail.com (M.C.B.); nicoleta2006r@yahoo.com (N.D.); roxanarusu12@yahoo.com (A.R.G.-R.); dr.popescu.diana@gmail.com (D.P.); ciprianrezus@yahoo.com (C.R.); 2 Internal Medicine Clinic, “Sf. Spiridon” County Clinical Emergency Hospital Iasi, 700111 Iasi, Romania; 3 Internal Medicine Clinic, Emergency Military Clinical Hospital Iasi, 700483 Iasi, Romania; 4 Department of Rheumatology and Physiotherapy, “Grigore T. Popa” University of Medicine and Pharmacy, 700115 Iasi, Romania; 5 I Rheumatology Clinic, Clinical Rehabilitation Hospital, 700661 Iasi, Romania

**Keywords:** microRNA, miRNA, miR, acute coronary syndrome, STEMI, myocardial infarction, MI, cardiovascular diseases

## Abstract

Regardless of the newly diagnostic and therapeutic advances, coronary artery disease (CAD) and more explicitly, ST-elevation myocardial infarction (STEMI), remains one of the leading causes of morbidity and mortality worldwide. Thus, early and prompt diagnosis of cardiac dysfunction is pivotal in STEMI patients for a better prognosis and outcome. In recent years, microRNAs (miRNAs) gained attention as potential biomarkers in myocardial infarction (MI) and acute coronary syndromes (ACS), as they have key roles in heart development, various cardiac processes, and act as indicators of cardiac damage. In this review, we describe the current available knowledge about cardiac miRNAs and their functions, and focus mainly on their potential use as novel circulating diagnostic and prognostic biomarkers in STEMI.

## 1. Introduction

There is an increased incidence and prevalence of atherosclerosis and coronary artery disease (CAD), which prolongs ischemic heart disease as one of the main causes of death worldwide [1]. Destabilization and afterward rupture of arterial plaque can produce acute coronary syndrome (ACS), which is classified into unstable angina, ST-segment elevation myocardial infarction (STEMI), and acute myocardial infarction (AMI) [2]. Accordingly, to the latest and fourth universal definition of myocardial infarction (MI), myocardial injury is defined as a different disease entity along with myocardial infarction [3]. STEMI is irreversible myocardial injury due to prolonged ischemia, and as the saying ‘time is muscle’ remains relevant, early and rapid diagnosis of MI still represents an upmost priority. Therefore, up-to-date cardiology guidelines highlight the pivotal need of rapid and early diagnosis and appropriate recovery of coronary flow, using primary percutaneous coronary angiography (PCI) or fibrinolytic therapy [4]. 

Because of the variable forms of debut, the first response at nitrate administration, which was used for many years as a diagnostic pointer, proved less reliable as an indicator of STEMI. Currently, the diagnosis is based on clinical symptoms, electrocardiogram modification, imaging evidence of cardiac cells ischemia, and circulating biomarker-level change [5]. To give a short cardiac marker history, the first cardiac–protein biomarker discovered and used in the 1980s and 1990s was creatine kinase myocardial band (CK-MB) [6]. Then, in the early 2000s, cardiac troponins I/ T (cTns I/T) were introduced as complementary to CK-MB, and soon after they were named as the new standard biomarker for acute cardiac injury [7]. Since then, cTns continue to represent the gold standard for MI diagnosis. However, even if they are still among the most widely used cardiac biomarkers, their low positive predictive power and low disease specificity can lead to incorrect diagnosis, as they are not completely specific to MI [8,9,10]. Additionally, the improved sensitivity of high analysis assay cTns (hs-Tns) is associated with prolonged time to correct diagnosis, undue interventions, and has diminished value in the first era of disease development, and its prognostic role is less well-established. The downside of this advancement is also an increased probability of false positive results, especially in the elderly population due to frequent associated comorbidities, which further emanate the need for new definition of acute pathological values in chronic diseases [11,12,13].

Thus, researchers are currently investigating new possible effective biomarkers for STEMI diagnosis with use in clinical practice [14,15,16]. In consideration of their roles, steadily growing research in life science of the expression pattern of cardiac tissue reveals that microRNAs (miRNAs/miR) are up- or downregulated during myocardial injury, showing their potential as biomarkers for AMI and ischemia–reperfusion injury (I/R) [17,18]. Accordingly, the gamut of new evidence lays out the potential role of miRNAs as novel biomarkers in acute and chronic cardiovascular diseases such as stable CAD [17,19,20,21,22], acute coronary syndromes (NSTEMI/STEMI) [23,24,25,26], or heart failure (HF) and cardiac remodeling secondary to MI [27,28]. 

With this review, we hope to convey a better image behind miRNAs and their role in myocardial infarction with predilection in STEMI disease; we discuss their potential predictive and prognostic roles and future use as biomarkers for early assessment of MI in clinical practice.

## 2. MiRNAs Superfamily 

Noncoding RNAs (ncRNAs) such as miRNAs are currently under investigation as potential additional or alternative biomarkers in cardiovascular diseases, showing promising results for implementation in clinical research [25,29,30]. The newly discovered long noncoding RNAs (lncRNAs) and circular RNAs (circRNAs) have also circulating marker features; however, their research is still at the beginning [31,32].

MiRNAs are endogenous noncoding short single-stranded RNA of 19–24 nucleotides in length that negatively regulate post-transcriptional gene functions. Over 2000 miRNAs have been identified in the human genome; they can target almost 60% of all genes which modulate the expression of around one third of all genes [21,33]. The structure of miRNA is delineated by their particular nucleic acid sequences; it is highly preserved in humans, animal, viruses, and plants, being first described in the 1990s during a study of *Caenorhabditis elegans*, a free-living transparent nematode [34]. They are intracellularly expressed and liberated extracellularly into plasma, saliva, breast milk, and urine, and are transported by blood cells, apoptic bodies, exosomes, lipoproteins, micro vesicles, or are connected with protein complexes [29,35]. Their vigorous stability to temperature changes and their resistance to degradation by endogenous RNase activity renders miRNAs as easy-to-use biomarkers in translational investigation and new tools for cardiac disease diagnosis [36,37]. 

Circulating miRNAs act as posttranscriptional regulators of gene expression by binding to the 3untranslated region (UTR) of the target gene, destabilizing the mRNA, translation repression, and thereby inhibiting protein synthesis/translation. They participate in many physiological and pathophysiological processes such as: the regulation of signaling platelet pathways, gene regulation of eukaryotes, angiogenesis, and insulin secretion [37,38,39,40]. MiRNAs are considered targets for personifying intervention and translational therapy [41]. Under pathological conditions, cells can usually passively or actively deliver microRNAs into circulation. In the midst of disease development, the characteristics of plasma, tissue, and cell miRNA change, forming a specific profile similar to a “fingerprint” for prediction, diagnosis, and prognosis [42,43]. Cardio-enriched miRNAs, such as miR-1, miR-195, miR-133, miR-126, miR-16, miR-590, miR-199, miR-143, miR-208a, miR-499, miR- 27-b, miR-497, miR-126, miR-30-d, miR-208b, miR-15a/b, and miR-16-1/2, take part in the regulation of cardiovascular system development [44]. Among them, miR-1 and miR- 133, which have the highest expression levels in the heart, have controversial tasks on cardiac cells as they promote cells proliferation and also inhibit cardiac differentiation [45]. On the contrary, miR-499 and miR-208 are found in lower concentrations in the heart; they are more specific in cardiac injury than in skeletal muscle [46]. 

**Figure 1:**
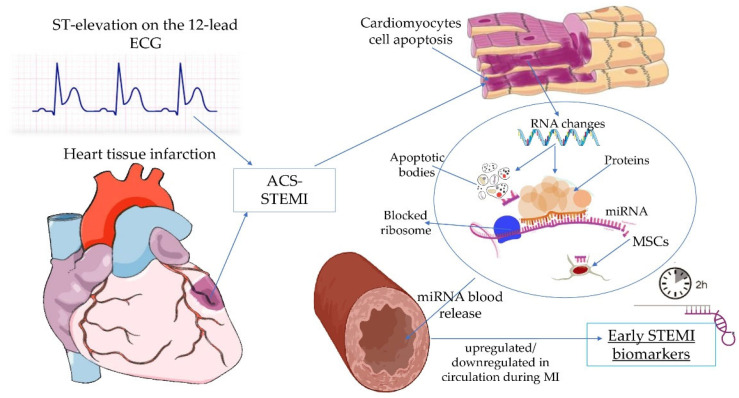
The generation and delivery of miRNAs in circulation during STEMI. In response to myocardial infarction, impaired cardiomyocytes release circulating miRNAs via protein complexes, microvesicles, exosomes, apoptotic bodies, and/or by mesenchymal stem cells, and then are either upregulated or downregulated. ST segment (ST); electrocardiogram (ECG); acute coronary syndrome (ACS); ST-segment elevation myocardial infarction (STEMI); ribonucleic acid (RNA); microRNA (miRNA); mesenchymal stem cells (MSCs).

Given that miRNAs can upregulate gene expression by binding to the promoter regions and target sites, many miRNA expression signatures are involved in oxidative stress, inflammation, apoptosis, fibrosis, and cardiac remodeling of ACS-related pathways [47,48,49,50,51,52,53,54,55,56,57,58,59,60,61,62,63,64,65,66,67,68,69,70] (Table 1). Nonetheless, every individual miRNA has its own particular role in cardiac biogenesis and progression, ergo, burgeoning scientific research which explores the role of miRNAs as novel diagnostic, prognostic biomarkers, and possible therapeutic targets in MI (Figure 1), is eagerly desired [71,72].

## 3. miRNA in Myocardial Infarction and STEMI 

miRNAs signatures were described having a role in MI for the first time more than a decade ago [73,74]. Since then, evidence points out that miRNA levels in the plasma of healthy subjects are almost undetectable, whereas in individuals with STEMI, a significant increase was measurable one hour after the onset of ischemia. Additionally, patients with suspected ACS had significantly increased levels of miRNAs, even in patients with initially negative troponin [75,76,77]. Therefore, many known miRNAs, especially, miRNA-1, miRNA-133 (both miRNA- 133a and miRNA-133b), miRNA-208, miRNA-208a, and miRNA-RNA 208b, are still being investigated in cardiac infarction [76,77,78,79,80]. 

### 3.1. miRNA-1, and miRNA-133

MiRNA-1 is one of the most highly conserved and expressed muscle-specific miRNA, which has two members, miRNA-1-1 and miRNA-1-2, which form bicistronic clusters with miR-133 [81]. Studies in embryonic stem (ES) cells reveal the role of miRNA-1 and miRNA-133 in driving cardiac differentiation. Cardiac-specific overexpression of miR-1 in the embryonic heart inhibits cardiomyocyte proliferation and prevents expansion of the ventricular myocardium [82]. Evidence shows that high levels of miR-133a and low levels of miR-1 seem to attenuate ischemic reperfusion injury [83], while increased microRNA-1 and microRNA-133a levels in the sera of patients with cardiovascular disease indicate the existence of myocardial damage [84]. Circulating miR-1 at admission showed incremental value in predicting left ventricle (LV) remodeling visualized with cardiac magnetic resonance, with an area under the curve (AUC) value of 0.68 (95% CI: 0.56–0.78), 6 months after STEMI [85]. Upon investigating patients undergoing transcoronary ablation of septal hypertrophy, other authors noted that plasma levels of miR-1, miR-133a, and cardiac-enriched miR-208a were raised in the first 4 h of cardiac injury. This increase also correlated with cTn levels; however, even if temporal release of ncRNAs may differ, a combination of these biomarkers could act as diagnostic tools [86]. 

MiRNAs such as miR-1, miR-126, miR-223, miR-199, and miR-21 are highly expressed in activated platelets by ischemia, therefore antiplatelet medication is affecting their plasma levels. While some suggest that they may have not diagnostic value, they may, however, play a predictive role in cardiovascular disease risk assessment [87,88]. Levels of platelet miR-1, with an arrive peak level within the first 2 h after the start of myocardial ischemia, seem to correlate with serum CK-MB concentrations [89]. In another research, circulating cardiac-specific miR-1 emerged as a marker of cardiomyocyte injury and loss of myocardial contractility, whereas endothelial-specific miR-126 concentration reflected endothelial activation and damage in the most extreme stage of atherosclerosis, and in the acute phase of AMI. Their values decreased after a follow-up period of 19.2 weeks [90]. Plasma levels of miRNA-133a and -133b, which peaked at approximately 2 h after occurrence of MI in STEMI [56], and circulating miR-122-5p/miR-133b ratio [91], may become specific early prognostic markers in acute MI. MiR-1 predicted left carotid artery stiffness along with miR-122, miR-132, and miR-133 in subclinical aortic atherosclerosis associated with metabolic syndrome [92], and miR-1, miR-208a, miR-133a, and miR-499 were found highly expressed in MI [93]. Both studies imply that when combined, these markers may have a more substantial diagnostic or prognostic value than any single miR, and future follow-up studies are needed to establish their clinical relevance. Moreover, a recent study revealed that miR-133b could significantly differentiate patients with STEMI from non-STEMI, and that he and miR-21 could become possible candidates of novel biomarkers in early prediction of CAD [94]. 

Among other studies, researchers noted that miRNA-1 is also a potential marker of cardiac injury in cardiogenic shock and is related to circulating glucose in STEMI patients, while miRNA-124a and -133 are more specific markers of STEMI. In spite of these results, they concluded that none of the miRNAs could be correlated to the extent of injury, progress of the disease, or prognosis of patient outcome, and therefore have no potential in becoming biomarkers of myocardial damage [95]. 

### 3.2. miRNA-208

Interestingly, miR-208a is cardiomyocyte-specific, not expressed by leucocyte, and may have a key role in cell proliferation and migration [96]. Research shows that miR-208 is associated with long-term prognosis following MI [97] and can be an independent predictor of the no-reflow phenomenon in STEMI individuals undergoing primary coronary intervention [98]. In a small size study, miRNA-208a was superior to cTnT in predicting occurrence of in-hospital major adverse cardiac events (MACE), in MI diagnosis, and in predicting outcomes of PCI-STEMI patients. The diagnosis performance of this microRNA is comparable to the known used cardiac biomarkers: CK-MB, cTnT and to hs-cTnT (*p* = 1.000). Authors believe that miRNA-208a is significantly better than routine biomarkers and is more specific and reliable than miR-30e [99]. miR-208b was considerably raised in the AMI subjects compared with healthy people, whereas miR-26a and miR-191 were decreased [100]. Release kinetics of circulating miRNA-208a were also observed in early phases of MI, the peak being registered at 3 h after reperfusion (*p* < 0.001), while traditional biomarkers such as cTnI and CK-MBmass reached the maximum concentrations at 6 h after reperfusion [101]. Moreover, peak values of miRNA-208b were well associated with the ejection fraction and troponin I levels [102], and a recent meta-analysis identified a significant association between miR-208 and mortality after AMI (HR 1.09, 95% CI 1.01–-1.18) [103]. Cheng et al. [104] found that urine concentration of miRNA-1 was raised and peaked at 24 h in rat model, and in STEMI patients, 60% of them had increased levels, whereas urine miRNA-208 can be found in only 25% of patients, suggesting that urine miRNA-208 might not be an applicable biomarker. Additionally, the early expression of miR-423-5p in AMI is significantly increased with subsequent normalization within 6 h, but levels of miR-1 and miR-208a were not significantly different in the STEMI group than in the control group, and no significant correlations between the expression level of miRNAs and any of the echocardiographic parameters of LV were found [105]. These conflicting results can be attributed to the sample size of the studies, quantification method, and probably due to pre-analytical and methodological variances.

### 3.3. Other miRNAs

miR-1-3p, miR-19b-3p, miR-208a, miR-223-3p, miR-483-5p, and miR-499a-5p are promising biomarkers for AMI due to their satisfactory diagnostic accuracy and short time window (within 4 h of the onset of symptoms) [106]. Circulating miR-19b-3p, miR-134-5p, and miR-186-5p could also be considered promising novel diagnostic biomarkers for the early phase of AMI [107]. Another study noted that miR-3113-5p, miR-223-3p, miR-499a-5p, and miR-133a-3p may provide independent diagnostic biomarkers for sudden cardiac death (SCD), a combination of two miRNAs presented higher diagnostic value (AUC = 0.7407–0.8667), and they could be further used to discriminate the causes of SCD [108]. After evaluating 66 AMI patients, the receiver operating characteristic (ROC) analysis indicated that miR-22-5p showed considerable diagnostic efficiency for predicting AMI, while plasma miR-22-5p levels were significantly decreased in these patients. Combining miR-122-5p and miR-22-5p raised the sensitivity (98.6%) while distinguishing patients with AMI and healthy comparisons [109]. Another report found significantly higher levels of miR-22-5p and miR-150-3p during the early stage of AMI, and that their expression levels peaked earlier than cTnI. In this case, miR-150-3p was the only miRNA investigated that was downregulated by medications for CAD and a combination of these three miRNAs improved diagnostic efficacy [110]. 

Lower levels of miR-26a and miR-191 were found in the plasma of individuals with acute MI [111]. Using cDNA synthesis and quantitative PCR, plasma levels of miR-21-5p and miR-146a-5p were significantly elevated in patients with ACS [22]. Expressions of miR-30d-5p, miR-146a-5p, and miR-23a-3p were statistically lower in patients with STEMI compared with the control group patients. Downregulation of miR-23a-3p was significantly negatively correlated with risk scores of APACHE II (Acute Physiology and Chronic Health Evaluation II) and GRACE (Global Registry of Acute Coronary Events), promoting this miRNA as potential new useful marker to assess short-term prognostic value and the severity of STEMI [112]. ROC analysis indicated that miR-126-5p, miR-145-3p, and miR-17-5p displayed more accurate diagnosis of AMI after PCI [113]. It is supposable that circulating miR-126, miR-197, and miR-223 levels are influenced by antiplatelet therapy in secondary prevention. Consequently, higher levels of microRNAs may mirror less-efficient platelet inhibition [114].

Validated by quantitative PCR, the expression of miR-155, miR-145, and let-7c was markedly reduced in patients with CAD compared with controls [115], while others exhibited that miR-486 and miR-150 plasma levels were significantly higher in AMI patients compared with healthy controls [116]. This current clinical trial (NCT03984123), which evaluates patients within the 48 h of STEMI post-PCI, noted that compared with baseline there were increased levels of miR-150,-21,-208 (*p* < 0.05) and reduced malondialdehyde after one or two cycles of bilateral brachial cuff inflation. Additionally, increased concentrations of miR-144 were related to the carotid-femoral pulse-wave velocity reduction (r = 0.763, *p* < 0.001) after the first cycle inflation [117]. 

Overexpression of miR-486-5p reduced cardiomyocyte apoptosis and improved cardiac function in rats by activating the phosphatidylinositol 3-kinase (PI3K)/ protein kinase B (Akt) pathway [118]. In another study, miR-122-5p was the only miRNA to be meaningfully upregulated in the sera of both patients with stable CAD and unstable CAD [119]. It is noteworthy that miR-142-3p might be an independent predictor of no-reflow during PCI in patients with STEMI [120]. After evaluating STEMI subjects treated with PCI who underwent cardiovascular magnetic resonance (CMR) imaging at 1 week and 6 months after STEMI, analysts discovered that miRNA-1254 predicted changes in left ventricle volumes and left ventricle ejection fraction (LVEF) at 6 months after STEMI [121]. QRT-PCR showed that plasma miR-941 level was elevated in the STEMI and the ACS group compared with the stable angina (*p* < 0.01) and NSTEMI groups (*p* < 0.05) [122]. MiR-663b, along with a signature consisting of several other miRNAs, has high statistical power in AMI with an accuracy of 92.5% [123]. Using kinetic analysis, the study discovered a fast time-dependent increase in miR-133a, miR-133b, miR-193b, miR-499, and miR-320a in STEMI at admission and after revascularization (at 3, 6, 12 and 24 h). Among them, only miR-320a was significantly associated with left ventricular (LV) adverse remodeling [124]. 

In this recent prospective observational study, authors objected a significant increase in miR-423-5p and miR-320a at 12 h compared with baseline (*p* < 0.001), with a notably decreasing levels from 12 to 24 h. As this is a small group study, results should be interpreted with caution; nonetheless, they underly the dynamic behavior of miRNAs (miR-21, miR-122, miR-320a, and miR-423-5p) during the first 24h of the coronary event [125]. The microarray analysis revealed miR-185 levels at discharge were significantly correlated with the troponin-I and CK-MB values, and one month after STEMI they were associated with a high wall motion score index and a low ejection fraction [126]. Li et al. [127] tried to find the role of pmiRNAs in myocardial pathogenesis. They noted that STEMI subjects had raised circulating levels of pmiR-150 and pmiR-223 and decreased levels of pmiR-126. Among them, only pmi-R126 presented a correlation with plasma troponin I, showing its potential as a novel biomarker for STEMI. In the same manner, pmiR-126 displayed positive correlation with cTnI (*p* = 0.011); however, they concluded that its diagnostic value is limited and more studies are needed. Other results exhibited that miR-126-3p along with miR-223-3p are promising independent predictors of thrombotic events and can be used for ischemic risk stratification after AMI [128]. 

#### 3.3.1. miRNA-30 and miRNA-146

Both miRNA-145 and miRNA-30c correlated with the size of myocardial tissue infarction [129]. Using PCR, authors identified lower plasma miRNA-30e levels at admission, which was an independent predictor of no-reflow in STEMI patients who underwent PCI, positively correlated with LVEF, and negatively correlated with high-sensitivity CRP levels [130]. Scientific research on rats showed that MiR-30e-3p is also involved in myocardial injury induced by coronary microembolization via autophagy activation [129], and that MiR-30e was poorly expressed in myocardial tissues of MI rodents [131]. When evaluating 89 STEMI patients with PCI, the low-sCD40L group had 3-fold higher levels of miR-19b at admission compared with the healthy group, and higher miR-145, miR-19b, and miR-222, at day 30, compared with stable angina patients [132]. Additionally, circulating concentration of miR-145 was correlated with infarct size, cTnI and CK-MB levels, and showed value as an independent predictor of cardiac events [133].

MiRNA-146a is considered a dominant negative regulator of innate immune response by negative feedback regulation of TRL signaling. Raised expression of this miRNAs indicates excessive inflammation [134]. Concentration of circulating miR-146a, miR-21, CK-MB, cTnI, NT-proBNP, as well as higher eGFR, were markedly higher in subjects with, than in those without of LV remodeling (*p* < 0.05) after STEMI. Interestingly, only the combination of miR-146a and miR-21 were independent predictors of LV remodeling [135]. One study reveals that miR-146a-5p is significantly elevated in patients with ACS [22], while on the contrary, miR-146a-5p was also detected statistically lower in patients with STEMI compared with the control group patients [112]. 

#### 3.3.2. miRNA-449a

Interestingly, miR-449a is one of the miRNAs that has protective effects against ischemia/reperfusion-induced apoptosis by inhibiting calcineurin-mediated dephosphorylation of dynmin-related protein-1. Knockdown of miR-499 persuaded myocardial apoptosis and elevated the infarct size [136]. Increased levels of miR-449a are correlated with AMI in rodent models and in humans [137], especially in the first days, followed by a decline to undetectable levels and with a sensitivity (0.84 (95% CI: 0.70–0.92)) and specificity (0.97 (95% CI: 0.87–0.99)) in AMI detection [103]. Upon evaluating patients with stable CAD and STEMI vs. control, authors found after adjustment for risk factors elevated miR499-5p levels and pointed out its role as an independent predictor of STEMI [138]. In a small sample study of the Egyptian population, AMI patients had higher expressions of the miR-499a (>105-fold, *p* < 0.001) variant, compared with hypertensive patients and healthy controls [139]. All 77 evaluated STEMI patients had significantly higher levels of miR-499-5p, miR-133a, and miR-133b [140]. Hs-cTnT in combination with miR-499, miR-1, or miR-21 achieved significant higher diagnostic performance than hs-cTnT alone [75]. 

#### 3.3.3. miRNA-20 and miRNA-26

The very recent Japan Collaborative Cohort Study for Evaluation of Cancer Risk (JACC) explored the potential relationship between circulating miRNAs and the risk of premature death. They discovered that miR-21 and miR-29a individual levels had a significantly higher risk of total, cancer, and cardiovascular disease (CVD) death than those with medium miR-21 and miR-29a levels. These results imply that miRNAs could be used as biomarkers for early detection of high-risk individuals of cancer and CVD [141]. In a secondary prevention framework performed on almost 900 patients diagnosed with CAD, only elevated miR-12+ showed predictive properties for future CV death [142]. MiR-21 is elevated in ACS [75], and has been associated with cardiac injury and also with cardio protection [87,94,143]. Its upregulation can attenuate cardiomyocyte apoptosis, the death of ischemic cortical neurons, and it may induce cardiac hypertrophy and fibrosis [144]. 

miR-26 is noted to participate in the pathology and recurrence of MI, by regulating miRNAs and other transcription factors which co-mediate this underlying processes. This micro-RNA could be measured in patients as an indicator of acute myocardial infarction [145]. In this small study, patients with STEMI and oxygen-glucose deprivation (OGD), had increased levels of creatine kinase (CK), creatine kinase-MB (CK-MB) and troponin I associated with miR-26a downregulation. They also found lower circulating levels of miR-26a in the infarct zone of the heart in comparison with the border and remote zones, in STEMI-induced mice at day one [146]. In contrast, it was previously described that miR-26a expression is raised in human patients with ACS and not lowered [147]. In conjunction, these data imply that the levels of miRNAs such as miR-26a are dynamically regulated via pathological mechanisms and different stimuli, at each stage of MI, acute, subacute, and chronic phase. 

#### 3.3.4. miRNA-155

miR-155 is upregulated in activated inflammatory cells, and it can modulate immune responses through cell differentiation and cytokine cell generation [148]. Over the years, many conflicting results regarding miRNA-155 have come forth [71]. One study described that miRNA-155 expression is highly elevated in human muscle tissue after ischemia–reperfusion injury, levels which were correlated with increased expression of TNF-α, IL-1β, and leucocyte infiltration. The same research highlighted in MI-rodents how miRNAS-155 aggravates the inflammatory response via modulation of suppressor of cytokine signaling 1 (SOCS-1)-dependent generation of reactive oxygen species. RNA silencing of the direct miR-155 target gene SOCS-1 abrogated this effect, showing its potential as a future therapeutic target [149]. MiRNA-155 seems to predict cardiac death within one year following hospital discharge for acute MI [150]. Impaired downregulation of its expression at day 5 was linked to subsequent adverse LV remodeling to STEMI, and it is positively associated with the monocyte (day 5, *p* = 0.046) levels [151]. Based on the result of this small sample study, we predict that a circulating biomarker such as miR-155 may be able to predict this STEMI consequence and help instate preventive measures that target inflammation at an early stage and could improve prognosis. 

#### 3.3.5. miRNAs in Plaque Vulnerability 

ROC curve analysis showed that a combination of these 3 miRNAs, miR-744-3p, miR-330-3p, and miR-324-3p, is associated with plaque rupture (PR) in STEMI patients compared with control. They may have clinical utility as diagnostic markers for categorization of plaque phenotype in STEMI, as independent predictors of PR, and for discriminating between patients with PR and patients with plaque erosion [152]. Notably, they are considerably enriched in the metabolism of bile, insulin, and thyroid hormone pathways, which are associated with plaque vulnerability [153]. Moreover, a clinical study investigated patients who underwent carotid endarterectomy and noted that miR-330-5p is associated with carotid plaques instability [154]. Other studies related to plaque vulnerability displayed evidence in which high levels of miRNA-3667-3p are linked to coronary plaque erosion in STEMI, and that MicroRNA-331 [155] and microRNA-151-3p could be novel biomarkers in STEMI caused by plaque rupture [156]. Notably, miR-324-3p seemed to raise the expression of insulin-like growth factor 1 (IGF1R), which was also associated with plaque instability [157], and levels of levels of miR-324-5p in endothelial progenitor cells derived from the peripheral blood of STEMI individuals were significantly lower compared with the healthy volunteers [158]. Interestingly, evaluation of one-year outcomes in hyperglycemic STEMI patients subjected to thrombus aspiration before primary PCI showed that hyperglycemic thrombi have resulted in increased miR33 expression and lower sirtuin 1 SIRT1, a member of the silent information regulator. This data points out the involvement of the miR33/SIRT1 pathway in the highly pro-coagulable and pro-inflammatory state coronary thrombi in hyperglycemic STEMI individuals [159]. Additionally, recent data show that miRNA-9 overexpression inhibits vulnerable atherosclerotic plaque formation and enhances vascular remodeling in the mouse model of acute coronary syndrome (ACS) [160]. Their involvement in plaque instability opens new pathways in visualizing miRNAs as possible therapeutic targets to prevent plaque rupture and, subsequently, the onset of ACS. 

## 4. Prognostic Role of miRNA in MI 

We previous described the involvement of miRNAs in atherosclerosis, CAD, plaque formation, erosion, and rupture. Some miRNAs could be a possible promoter for HF and other adverse clinical outcomes. They not only exhibit a predictive role in cardiovascular events, but also can mediate post-MI events, with substantial value in appreciation of ACS prognosis. Studies targeting miRNAs have investigated their prognostic function concerning the ability to predict left ventricular (LV) remodeling and cardiovascular mortality [61,112,139,161,162]. 

Upon analyzing, researchers have found that patients with low levels of miR-101 or miR-150, and elevated levels of miR-16 or miR-27a, were at higher risk of flawed LV contractility after STEMI [163], and that miR-150 has a strong individual relationship with post-MI LV remodeling [164]. MicroRNA-133a and miR-133b were also evaluated in STEMI prognosis [165], being positively associated with microvascular obstruction and worse LV functional recovery [166]. MicroRNA-133a concentrations showed significant correlations (*p* < 0.001) with all prognostic factors detected by CMR (infarct size, microvascular blockage, myocardial salvage index). Even if major acute cardiovascular events (MACE) occurred significantly more often in the miR-133a group, its concentrations were unable to independently predict clinical events [167]. Others showed that the miR-122-5p/133b ratio may be a new prognostic biomarker for the early identification of STEMI patients at a higher risk of developing MACE after undergoing PCI intervention [91]. While varied levels of miR-184 showed a positive correlation with MACE [168], miR-192, miR-194, and miR-34 were significantly higher in the sera of patients who later developed HF [169], both results displayed the promising role of these miRNAs as prognostic biomarkers in MI. 

This multicenter, prospective SPUM-ACS-Cohort showed that miR-26b-5p, miR-320a, and miR-660-5p are associated with adverse cardiovascular outcomes in STEMI subjects, discriminated for MACE, and increased risk prediction when added to the Global Registry of Acute Coronary Events (GRACE) score. For the first time, researchers performed a miRNA profiling and validation approach to assess miRNAs related to adverse prognosis in MI [170]. Related to prognosis, raised levels of miR-133a, miR-208b, miR-197, and miR-223, were strong predictors of the risk of cardiovascular death in patients with ACS [142], and miR-208b, miR-34a, and miR-499-5p were highly associated with increased risk of death/HF in MI subjects [171]. In addition, miR-1 and miR-499 presented high accuracy in discriminating sudden cardiac death from AMI [172] while, elevated circulating miR-328 and miR- 134 levels were correlated with a high risk of death or HF within 6 months of AMI [173]. Other miRNAs such as, circulating miR-30a-5p and miRNA-148 exhibit potential as a prognostic biomarker in MI. MiR-30a-5p levels predicts LV dysfunction and HF onset after acute MI [174], and miRNA-184 has a dynamic evolution before and after PCI treatment for AMI, being correlated with recent ventricular remodeling indexes and the future occurrence rate of MACE [168]. The large AtheroGene study concluded that single miRNAs could predict mortality in secondary prevention settings, improving various model performance measures, and can represent valuable biomarkers for risk estimation in ACS [175].

Considering the present data, high miRNA expression may be an independent risk factor for patients with MI and could be a promising prognostic biomarker for post-MI and implicitly STEMI, MACE and sudden cardiac death, assessment [162,176]. A systematic review and meta-analysis highlighted the barriers behind the use of miRNAs as prognostic markers and do not support use of miRNAs for prognostication post-ACS beyond traditional cardiovascular risk factors, stratification tools, and existing risk scores [78]; therefore, further scientific research and larger prospective studies using normalized tests for miRNA are warranted to validate our conclusion.

## 5. Discussion

Since they were first discovered, continuously and rigorous research tried to find the precise role of miRNA in different known pathologies including cardiovascular disease. As observed, they detain key functions in cardiac biogenesis, development, and progression, and given their stable structure and rapid circulation release after myocardial injury, they were proposed as potential future biomarkers in ACS. More specifically, older and recent evidence point out the potential role of miRNAs as novel biomarkers not only in STEMI, but also in STEMI’s secondary complication such as LVR or HF (Table 1), [13,14,15,16,17,18,19,20,21,22,23,24,25,26,27,28,29,30,31,32,33,34,35,36,37,38,39,40,41,42,43,44,45,46,47,48,49,50,51,52,53,54,55,56,57,58,59,60,61,62,63,64,65,66,67,68,69,70,71,72,73,74,75,76,77,78,79,80,81,82,83,84,85,86,87,88,89,90,91,92,93,94,95,96,97,98,99,100,101,102,103,104,105,106,107,108,109,110,111,112,113,114,115,116,117,118,119,120,121,122,123,124,125,126,127,128,129,130,131,132,133,134,135,136,137,138,139,140,141,142,143,144,145,146,147,148,149,150,151,152,153,154,155,156,157,158,159,160,161,162,163,164,165,166,167,168,169,170,171,172,173,174,175,176,177,178,179], or even their use as promising new therapeutic targets [180,181] (Table 2). 

More than that, miRNAs have the potential to be used in the differential diagnosis of ACS [17,182] with: unstable angina [183], NSTEMI [94,184], and acute myocarditis [185]. Exploration of patients with STEMI and patients with Takotsubo cardiomyopathy (TTC), established that miR-133a was substantially increased subjects with STEMI compared with those with TTC. A unique signature comprising miR-1, miR-16, miR-133a, and miR-26a, differentiated TTC from STEMI patients (AUC 0.881, 95% CI 0.793–0.968, *p* < 0.0001) with a sensitivity of 96.77% and a specificity of 70.37% [186]. MiR-1 and miR-133a have also been previously reported to be slightly alleviated in patients with unstable angina and TTC, while their expression is strongly upregulated in STEMI subjects [84]. Very recently, after investigating coxsackievirus-induced myocarditis mice models and a cohort of humans with myocarditis, STEMI, and NSTEMI vs. controls, authors identified the human homologue (hsa-miR-Chr8:96) that could be used to distinguish patients with myocarditis from those with MI [187]. The presented information layout the possibility of using miRNA analysis for the differential diagnosis of ACS. We must, however, interpret these results with caution, considering limitations of the studies, selection, small sample size, variable normalization of data, and adjustment for confounders. Additionally, anticoagulants, antiplatelet drugs, and other medication therapies affect plasma miRNA profiles [63,64,91].

It is well known that STEMI is mainly an electrocardiographic diagnosis; however, as miRNA are involved in atherosclerosis with subsequent plaque formation, erosion, and rupture [152,153,154,155,156,157,158,159,160], they perhaps would rather be more useful in the potential detection of vulnerable plaque as a generator of myocardial damage and STEMI. The levels of circulating miRNA are elevated early after the onset of chest pain when there is no upregulation in serum creatine phosphokinase or cTnT, and because some of the biggest advantages for using miRNAs as biomarkers is their stability and bioavailability [36,37], maybe an overreach future idea may include the development of rapid at-home devices that can scan a small blood sample to determine miRNAs levels, for prediction of near future acute cardiac events. Although the accuracy of a single miRNA in detecting cardiac injury is poor, a panel of multiple miRNAs or a combination with cardiac troponin may improve the diagnostic power. Besides their potential use as diagnostic biomarkers, researchers focus more on their potential as prognostic markers for adverse myocardial effects, sudden death, and risk assessment [163,164,165,166,167,168,169,170,171,172,173,174,175,176,177].

Overall, even if these biomarkers have shown good sensitivity for MI, most of them are missing the specificity and diagnostic efficacy in comparison with cardiac troponins. Current miRNA assays (RT-qPCR and microarray) lack sensitivity for early detection of miRNA and are currently quite expensive and extremely time-consuming, limiting the clinical use of the results. Blood-based immunoassay, which can be immediately integrated into standard diagnostic procedures, and grant a more sensitive detection and earlier rule-in and rule-out of myocardial injury, is desired.

Based on the current knowledge displayed so far, it is safe to declare that larger multicenter trials are required to establish whether they actually offer additional benefits over the existing diagnostic and prognostic biomarkers in ACS-STEMI.

## 6. Conclusions

While this field has already been extensively studied, a need to upgrade and complement existing biomarkers for CAD and ACS is imperative. With this purpose, we believe that miRNAs own predictive biomarker potential in atherosclerotic context and potential prognostic role in ACS such as STEMI. Perhaps a combination of new more sensitive miRNAs, in addition to cardiac troponins, could improve risk assessment of future acute cardiac events post-cardiac injury. Furthermore, refinement of current approaches and development of new protein assays and devices, that fasten and improves detectability of myocardial injury, could extend the range of early paraclinical diagnosis of MI, improve risk stratification, and long-term prognosis.

## Figures and Tables

**Table 1:** Different miRNAs and their influence on pathophysiological pathways involved in myocardial infarction or after infarction.

miRNAs	Cardiac Processes	Biological Pathways	Ref.
miR-378	Modulates cardiac fibrosis	PCFL regulates cardiac fibrosis via miR-378/GRB2 pathway;	[47]
miR-101	Cardiac fibrosis	By decreasing c-Fos and its downstream TGF-β1	[48]
miR-208a	Apoptosis	By Upregulating Bax;	[49]
miR-208a	Promoted apoptosis and oxidative stress	By regulation of protein tyrosine phosphatase receptor type G and protein tyrosine phosphatase; non-receptor type 4	[50]
miR-208a	Myocardial fibrosis	via upregulation of endoglin;	[51]
miR-208a	Cardiac hypertrophy and fibrosis	Via upregulation of endoglin after activation by TGF-β1;	[52]
miR-223	Cardiac fibrosis	By negatively regulating RASA1 expression, and it mediates the pro-fibrotic effects of TGF-β1 in vitro;	[53]
miR-133a	Apoptosis suppressor	By inhibiting TAGLN2, HSP60, HSP70, Apaf-1, caspase-3/8/9 expression, and promoting antiapoptotic protein Bcl-2 expression, and by regulating caspase-9.	[54]
miR-133a	Inhibits angiogenesis,	By targeting SRF;	[55]
miR-133a	Inhibits angiogenesis,	Via VEGFR2 and fibroblast growth factor receptor 1;	[56]
	Anti-apoptotic role	By inhibiting proapoptotic genes: death-associated protein kinase 2 (DAPK2), apoptotic protease activating factor 1 (APAF1), caspase-9, Bcl-2-like 11, and Bcl-2-modifying factor (BMF);	[57]
MiRNA-23a	Cardiac apoptosis	By suppressing the expression of manganese superoxide dismutase.	[58]
miR-26a-5p	Cardiac fibrosis	Regulation of cardiac collagen I expression by targeting ULK1;	[59]
miRNA-26b	Relieves inflammatory response	By suppression of mitogen-activated protein kinase (MAPK) pathway through binding to Prostaglandin-Endoperoxide Synthase 2 (PTGS2);	[60]
miRNA-144	Oxidative stress	Through regulation of Forkhead Box O1;	[61]
miRNA-24-3p	Reduces apoptosis	Via regulation of Keap1-Nrf2 pathway in response to ischemia/reperfusion injury;	[62]
miR-21	Attenuates inflammation	Through targeting kelch repeat and BTB (POZ) domain containing 7 and inhibiting p38 and NF-κB signaling activation;	[63]
miRNA-143-3p	Promotes fibrosis	By activation of P38, ERK, and JNK pathways;	[64]
miRNA27a, miRNA-28-3p, miRNA-34a	Contribute to oxidativestress	By the inhibition of Nrf2 translation in chronic heart failure post-MI; contributing to the dysregulation of the Nrf2/ARE signaling pathway;	[65]
miRNA-320	Cardiomyocyte death and apoptosis	By regulating small heat-shock protein 20 (Hsp20) protein synthesis;	[65]
miR-200a	Reduce inflammation	By targeting the Keap1/Nrf2 and β-catenin pathways;	[66]
miR-6391, miR-671, miR-558, miR-1538	Apoptosis in the non-infarcted areas after MI	Regulation of the proteins involved in the synthesis and signaling cascade of sphingolipids;	[65]
miR-6391	Tissue remodeling	Via regulation of the neurotrophin signaling pathway;	[67]
miR-25, miR-3535, miR-6391	Cardiac fibrosis	Via downregulation of collagen organization.	[68]

microRNA (miRNA); pro-cardiac fibrotic lncRNA (PCFL); growth factor receptor bound protein 2 (GRB2); myocardial infarction (MI); transforming growth factor-β1 (TGF-β1); bcl-2-associated X-protein (Bax); RAS p21 protein activator (GTPase-activating protein) (RASA1); serum response factor (SRF); vascular endothelial growth factor receptor 2 (VEGFR2); unc-51-like autophagy activating kinase 1; antioxidant response element (ARE); nuclear factor-erythroid factor 2-related factor 2 (Nrf2); Kelch-like ECH-associated protein 1 (Keap1).

**Table 2:** Current information on the role of various miRNAs in STEMI.

Subjects Enrolled	Animal	Detection Method	Marker Comparison	Salient Findings	Year	Ref.
33 STEMI vs. controls	C57BL/6 female mice	qRT–PCR	cTnI	-Upregulation of miR-1, -133a, -133b, and -499-5p plasma levels, both in humans and mice; -miR-122 and -375 lower than control only in STEMI patients;	2010	[56]
397 STEMI vs. 113 NSTEMI vs. 87 control	-	qRT–PCR	hs-cTnT	-A total of 3 h after onset of pain, miR-499 was positive in 93% of patients and hs-cTnT in 88% of patients (*p* -0.78); miR-499 and hs-cTnT provided comparable diagnostic value with areas under the ROC curves of 0.97;	2012	[93]
STEMI vs. control	Rat model of AMI	qRT–PCR	serum TnI	- A total 50-fold increase in miR-1 level in urine from rats at 24 h after AMI (*p* < 0.0001; in humans: a positive correlation serum TnI and urine miR-1 levels (r = 0.70; *p* < 0.05), 5 patinets had very low levels of miR-208 in urine;	2012	[104]
237 STEMI post-pPCI	-	qRT–PCR	CK-MB NT-proBNPcTnI	- miR-21 correlated with cTnI (*p* < 0.0001), but not with CK-MB (*p* = 0.064)/ NTproBNP (*p* = 0.0665); miR146a (odds ratio, OR = 2.127, *p* < 0.0001), miR-21 (OR = 1.119,*p* < 0.0001) predictors of LVR;	2015	[135]
77 STEMI, 21 NSTEMI vs. 23 control		qRT–PCR and ELISA	cTnI	- miR-133b and miR-499-5p were significantly higher in the early phase (the first 4 h) (*p* < 0.05);	2015	[140]
50 STEMI, 50 stable CAD vs. 50 control	-	qRT–PCR	-	- miR499-5p independent predictor of STEMI (OR = 3.03, *p* = 0.001); MiR15a-5p, miR146a-5p, and miR16-5p had AUCs of 0.67, 0.65, and 0.68, respectively;	2016	[139]
16 STEMI vs. 27 NSTEMI	-	qRT–PCR	-	- miR-134 s 3.83-fold higher in the STEMI with IRA occlusion group (*p* < 0.025); significantly higher hs-TnT levels, compared with NSTEMI;	2016	[178]
5 STEMI, 5 NSTEMI vs. 5 controls	-	qRT–PCR	-	- plasma miR-941 level was elevated 2.28-fold in STEMI compared with non-CAD (*p* < 0.05);	2017	[122]
20 STEMI vs. 8 control	-	qRT–PCR	-	- miR-155 (day 5) was higher in patients with adverse LVR, compared with patients without adverse LVR; its levels were associated to relative change in end-diastolic volume (ρ = 0.490, *p* = 0.028);	2017	[151]
9 STEMI, 5 NSTEMI vs. 12 controls	-	sRNA-seq and qRT–PCR	-	- miR-134-5p, miR-15a-5p, and let-7i-5p significantly downregulated (5-fold, 7-fold and 3.5-fold, respectively); discriminatory power was highest with let-7i-5p (AUC = 0.833);	2018	[76]
225 STEMI post-pPCI	-	qRT–PCR	hs-CRP	- miRNA-30e yielded AUC of 0.914 (95% CI: 0.870–0.957; sensitivity¼ 82.7%, specificity¼ 88.6%, *p* < 0.001)—independent predictor of the no-reflow phenomenon during pPCI STEMI patients;	2018	[98]
70 STEMI	-	qRT–PCR	-	- miRNA-1254 was associated with decreasing LVESVI (*p* = 0.006) and significant positive association with increasing LVEF during follow-up (*p* < 0.001);	2018	[121]
20 STEMI, 18 NSTEMI- TASH, vs. control	-	qRT–PCR	cMyBP-C hs-cTNI/T CK-MB CK	- miR-208b and miR-499(*p* < 0.0001) had the highest correlation with hs-cTnT; miRNAs failed to identify cases presenting with low troponin value;	2019	[25]
62 STEMI vs. 26 controls	-	qRT–PCR	-	- miR-30d-5p, miR-146a-5p, and miR-23a-3p were, respectively, 1.581-fold, 4.048-fold, and 4.857-fold lower in patients with STEMI (<0.001)	2019	[112]
40 STEMI	-	qRT–PCR	-	-miR-28a diagnostic accuracy for MI (AUC = 0.926); after primary PCI, miR-208a it was superior to cTnT in prediction of no-reflow (AUC difference of 0.231, *p* = 0.0233) and MACE (AUC difference of 0.367, *p* = 0.0053;	2020	[99]
80 STEMI	-	PCR	CK–MB NT-proBNP troponin T	-miR-1 expression predicted LV remodeling with AUC value of 0.68 (95% CI: 0.56–0.78);	2020	[85]
15 STEMI vs. 11 US/NESTEMI vs. 54 control	-	qRT–PCR	-	- Both miRNAs differentiated STEMI from NSTEMI with miR-133b AUC 0.80 with >75.6% sensitivity and specificity; AUC for miR-21 was 0.79 with >69.4% sensitivity and specificity;	2020	[94]
42 STEMI post-pPCI vs. 14 control	-	qRT–PCR	CK	- miR-29a, miR-29b, miR-324, miR-208, miR-423, miR-522, and miR-545 was differentially expressed before pPCI in STEMI; miR-320a as an independent predictor of LVAR (*p* < 0.045);	2020	[120]
270 STEMI post-pPCI	-	qRT–PCR	-	- Increased miR-150,-21,-208 (*p* < 0.05); raised miR-144 was related to PWV reduction (r = 0.763, *p* < 0.001);	2021	[117]
41 STEMI vs. 17 control	-	qRT–PCR	-	- miR-744-3p, miR-330-3p, and miR-324-3p distinguishing between PR and PE;	2021	[152]

qRT-PCR quantitative real time polymerase chain reaction (qRT –PCR); small RNA sequencing (sRNA-seq); CK-MB creatine kinase MB ; cardiac Troponin I (cTnI); high sensitivity troponin (hs-Tns); cardiac myosin-binding protein C (cMyBP-C); area under the curve (AUC); primary percutaneous coronary intervention (pPCI); transcoronary ablation of septal hypertrophy (TASH); infarct-related artery (IRA) occlusion; major adverse cardiac events (MACE); acute myocardial infarction (AMI); carotid-femoral pulse-wave velocity (PWV); left ventricular adverse remodelling (LVAR); lef ventricle end-systolic volume index (LVESVI); left ventricule ejection fraction (LVEF).
